# Vestibulotoxicity Associated With Platinum-Based Chemotherapy in Survivors of Cancer: A Scoping Review

**DOI:** 10.3389/fonc.2018.00363

**Published:** 2018-09-25

**Authors:** Pattarawadee Prayuenyong, John A. Taylor, Stephanie E. Pearson, Rachel Gomez, Poulam M. Patel, Deborah A. Hall, Anand V. Kasbekar, David M. Baguley

**Affiliations:** ^1^Hearing Sciences, Division of Clinical Neuroscience, School of Medicine, University of Nottingham, Nottingham, United Kingdom; ^2^NIHR Nottingham Biomedical Research Centre, Nottingham, United Kingdom; ^3^Nottingham University Hospitals NHS Trust, Nottingham, United Kingdom; ^4^Department of Otorhinolaryngology, Head and Neck Surgery, Faculty of Medicine, Prince of Songkla University, Songkhla, Thailand; ^5^Division of Cancer and Stem cells, School of Medicine, University of Nottingham, Nottingham, United Kingdom; ^6^University of Nottingham Malaysia, Semeniyh, Malaysia; ^7^Department of Otorhinolaryngology, Head and Neck Surgery, Nottingham University Hospitals NHS Trust, Nottingham, United Kingdom

**Keywords:** vestibulotoxicity, vestibular, adverse effect, platinum-based chemotherapy, cancer

## Abstract

**Background:** Cochleotoxicity following the treatment with platinum-based chemotherapy is well documented. The potential for vestibulotoxicity is still unclear. This scoping review examined the extent of current research literature, summarized research findings and identified research gaps regarding vestibular-related adverse effects associated with platinum-based chemotherapy in survivors of cancer.

**Methods:** Inclusion criteria followed the PICO principles: Participants, adult, and pediatric cancer patients of any cancer type; Intervention, platinum-based chemotherapy (such as cisplatin, carboplatin, and oxaliplatin); Control, none or any; Outcomes, vestibular-related adverse effects. English language articles published since 1978 were retrieved. Seventy-five eligible studies were identified from a systematic literature search, and relevant data were charted, collated, and summarized.

**Results:** Testing for vestibulotoxicity predominately featured functional evaluation of the horizontal semicircular canal using the caloric and rotational tests. The rate of abnormal vestibular function test results after chemotherapy administration varied from 0 to 50%. The results of objective testing did not always correspond to patient symptoms. There is tentative support for patients with pre-existing loss of vestibular function to be more likely to experience vestibular toxicity after dosing with cisplatin.

**Conclusions:** A number of studies reported significant evidence of vestibular toxicities associated with platinum-based chemotherapy, especially cisplatin. This scoping review emphasizes that vestibular toxicity needs more attention and comprehensive evaluation. Specifically, studies that analyse cumulative dose of platinum-based chemotherapy, affected sites of lesion in vestibular end organs, and the correlation and temporal patterns of cochlear and vestibular toxicity are needed.

## Introduction

More than 15.5 million cancer survivors are living in the United States alone, and this number is set to increase to 20 million by 2026 because of advances in early detection, effective treatment, and the aging and growth of the population (1). As a consequence of these increased survival rates, more attention is being given to improvements in long-term effects, health-related quality of life, and follow up care after cancer treatment (2).

Platinum-based chemotherapy is an effective antineoplastic intervention that is used for a variety of human malignancies including testicular, ovarian, bladder, head and neck, and non-small cell lung cancer (3). Irreversible hearing loss following the treatment with platinum-based drugs, especially cisplatin (which causes permanent hearing loss of variable degree in 40–80% of treated patients) is well documented (4). Long-term monitoring of cochleotoxic effects of platinum-based chemotherapy is therefore advised (5) and implemented in clinical practice (6). There is also some evidence that cisplatin has long-term retention in the cochlea (7). Given that the auditory and vestibular organs of the inner ear share the same blood, nerve and fluid supplies, this finding has potential implications for functions of both compartments (8). However, there are some differences in physiology and function between the cochlear and vestibular end organs, and these may mediate the impact of toxicity associated with platinum compounds. Specific areas of interest include the role of the stria vascularis in the cochlea (9), as no analogous structure is present in the vestibular labyrinth, and the possible role of transporters such as megalin (10). There is a strong potential for cochlear toxicity to be accompanied by vestibular toxicity in patients receiving platinum-based chemotherapy. However, clinical reports of inner ear toxicity are limited largely to auditory symptoms.

The vestibular organs of the inner ear play an important role in the complex and dynamic human balance system (11). The peripheral vestibular system in the inner ear consists of five sensory organs: three semicircular canals (horizontal, anterior, and posterior) and two otolith organs (saccule and utricle) on each side (11). The integration occurs at the central nervous system giving efferent fibers to vestibulo-ocular, and vestibulo-spinal pathways (11). No clinical test can directly measure the inner ear function of the vestibular periphery. Instead, inferences must be made about vestibular function on the basis of the performance of downstream processes especially the vestibulo-ocular reflex (VOR). In the wider context, the balance system covers the spatial orientation of the whole body, which is maintained by the integration of visual, somatosensory, and vestibular inputs to the central nervous system (11). While the vestibular function refers to the health of the vestibular portion of the inner ear, balance function is not restricted to a vestibular component alone.

The potential for vestibular toxicity is still unclear, though clinical data suggests it is problematic (12, 13). There are a number of potential explanations for why vestibulotoxicity from platinum-based chemotherapy is less frequently described than auditory symptoms. First, some of the clinical signs and patient-reported symptoms may be underappreciated by clinicians. Notably, drug-induced vestibular loss may affect both ears symmetrically and gradually resulting in insidious disequilibrium, postural imbalance, and oscillopsia (illusion of movement of the visible world during head movement). These symptoms are less likely to undergo clinical assessment, compared with the sudden onset of spinning vertigo such as benign paroxysmal positional vertigo (BPPV), vestibular neuritis and Meniere's disease (14, 15). Secondly, vestibular organ dysfunction may be masked by central compensation or substitution by vision and proprioception, obscuring vestibular damage compared with the more noticeable cochlear damage (16). Thirdly, platinum-based chemotherapy agents are used in combination with several other drugs, and some of these may have ototoxic properties such as aminoglycoside antibiotics (17) and loop diuretics (18). Thus, the specific attribution of vestibulotoxicity to platinum-based compounds may be obscured. Finally, non-specific symptoms of imbalance may be attributed to underlying cancer diseases and general deconditioning of patients during and after treatment such as dehydration, nausea and vomiting, chronic fatigue, and anemia.

Vestibular dysfunction can have a considerable impact on quality of life (19) and substantial economic burden on individuals and society (20). Recent evidence suggests that balance problems such as falls and mobility disability in survivors of cancer are more common than amongst the general population (21–23). This is of great importance as falling is a leading cause of morbidity and mortality in the community population (24). Therefore, there is a need to increase awareness of balance problems in this vulnerable group of cancer patients in order to provide accurate prevention and intervention measures (25).

While systematic review methodology seeks to collate all evidence in order to address a specific research question, scoping review methodology aims to map the key concepts underpinning a research area and the main sources and types of evidence available (26). A scoping review can be undertaken as a stand-alone project in its own right, especially where an area has not previously been reviewed in a comprehensive manner (26). This scoping review examined the extent of current research literature on vestibular-related adverse effects associated with platinum-based chemotherapy in survivors of cancer to summarize research findings and identify research gaps.

## Method

The method of this scoping review is largely based on the steps of the framework proposed by Arksey and O'Malley (26): (1) identify relevant studies, (2) select studies, (3) chart the data, (4) collate, summarize and report the results, and (5) consult clinical experts. Study details were registered on PROSPERO (CRD42017083576).

### Identify relevant studies

The following databases were searched: Medline, EMBASE, TOXLINE, IPA (International Pharmaceutical Abstracts), Science Citation Index-Expanded, ProQuest Dissertations & Theses A&I, ClinicalTrials.gov, Cochrane Central Register of Controlled Trials (CENTRAL), and International Clinical Trials Registry Platform (ICTRP). Gray literature was also considered through the Database of Adverse Event Notifications [Australia], Drug Safety Update [UK], European Public Assessment Reports via European Medicines Agency [Europe], and Medwatch [USA]. The steps followed the Cochrane Handbook (27) and the Cochrane Methodological Expectations of Cochrane Intervention Reviews (MECIR) (28) for conducting the search, the PRISMA guideline (29) for reporting the search, and the PRESS guideline for peer-reviewing the search strategies (30). The search strategy followed recommendations for optimizing the search syntax to identify adverse effects (31–34). Standardized terms and keywords were combined in the search for concepts of platinum-based chemotherapy and vestibular toxicity. Keywords were collected through expert opinions, literature reviews, controlled vocabulary (Medical Subject Headings = MeSH and Excerpta Medica Tree = EMTREE), and reviewing preliminary search results. Filters for electronic databases were applied where possible to retrieve articles in the English language, with human participants, and time of publication since 1978 which is the time that cisplatin, the first drug in the platinum-based chemotherapy group, was approved by Food and Drug Administration (FDA) for human use (3). We limited our search to the English language because of resource constraints. The search strategies are reported in Appendix 1.

### Study selection

Two screening steps were undertaken independently by two authors. The first step checked that each title and abstract was within the scope of research question. Examples of “out of scope” decisions were where there was no mention of platinum-based chemotherapy, *in vitro* or *in vivo* studies, or review articles or conference papers. The second step of full text screening considered eligible records that contained data pertinent to the subject of the review, specifically vestibular-related symptoms and/or test results in adults and/or pediatric cancer patients undergoing platinum-based chemotherapy (such as cisplatin, carboplatin, and oxaliplatin) in any cancer types (either alone or in combination with other treatments such as radiotherapy or surgery). Eligible studies were randomized controlled trials (RCTs), non-randomized controlled trials, observational studies, cross-sectional studies, cohort studies, case control studies, case series, and case reports. Discrepancies were resolved through discussion and a third reviewer was consulted whenever a consensus was not reached. Literature saturation was further accomplished by additional hand searching of the reference lists of all included studies and also those of the excluded review articles.

### Charting the data

Pre-specified data items included year, study design, participant demographics and characteristics, sample size, cancer treatment intervention, drug dose, and patient evaluation. These data items provided useful information about the scope and details of each record, enabling the authors to look for common themes and to identify possible gaps in the literature. Multiple reports pertaining to a single study were treated as one, but data extraction considered information presented in all records. Data from each included study was independently extracted by two clinical experts on the team (PP, an otorhinolaryngologist and DB, an audiologist). Discrepancies were identified and resolved through discussion.

### Collating, summarizing and reporting the results

For the purpose of understanding key concepts in this literature, thematic analysis was conducted following article review to summarize the literature into themes by the two members of the research team (PP and DB). The authors independently reviewed the charted final data set and identified themes, and then met to discuss possible thematic structures, using the criteria that themes should be important clinical aspects and should adequately represent all of the records. Specific themes identified were objective tests of vestibular and/or balance function, patients' symptoms, physical examination, associated factors, and general considerations. The authors then grouped all studies according to these themes. The content of individual records does not necessarily fall exclusively in one theme or another; hence, the records could contribute to more than one theme. Research findings were summarized and research gaps were identified.

### Clinical expert consultation

Two clinical experts on the team (AK, an otorhinolaryngologist and PMP, an oncologist) reviewed the themes and supporting evidence of the results.

## Results

**Figure 1:**
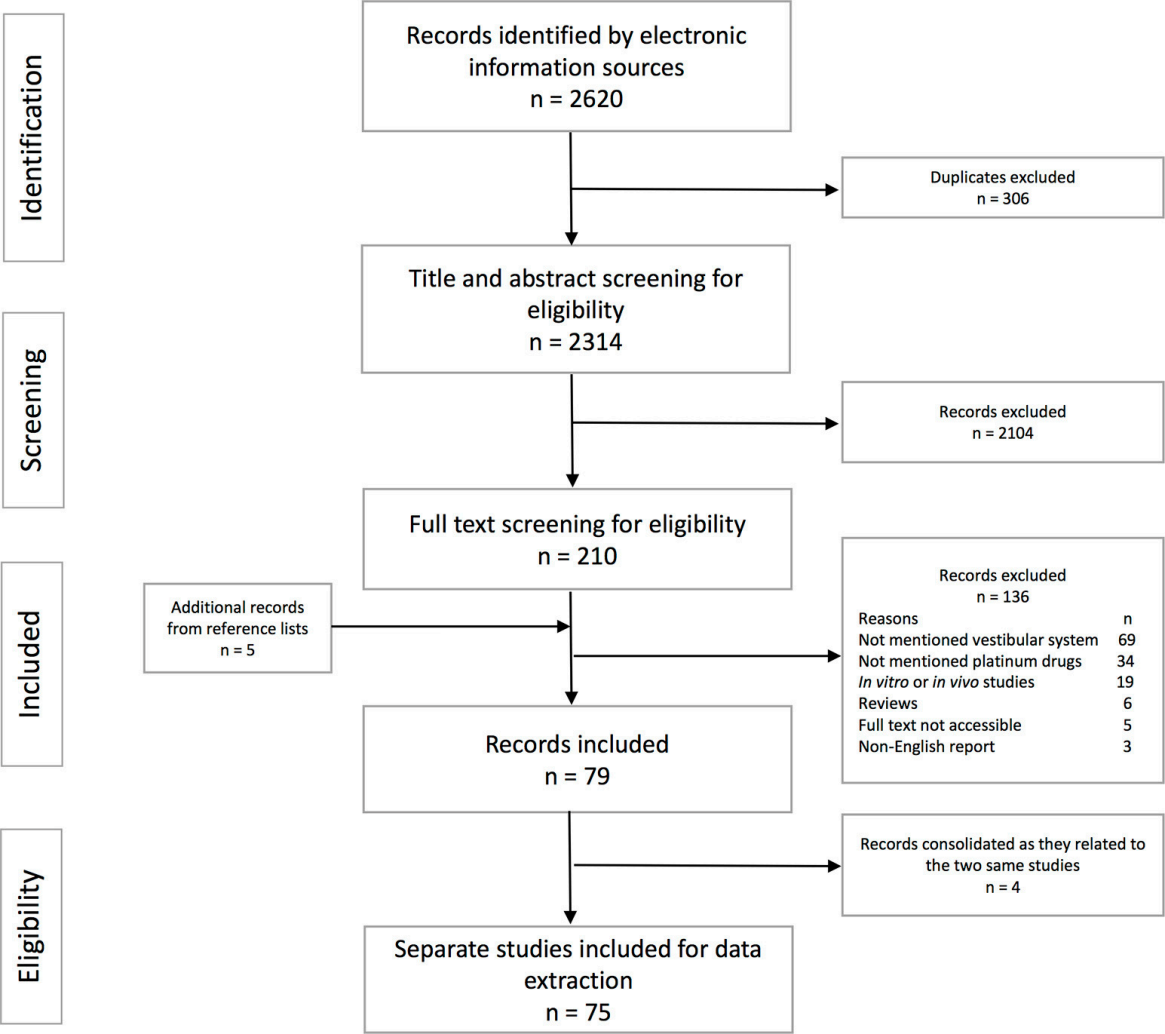
Flow chart of stages of the study selection process.

A summary of the study selection processes with the reasons for exclusion is represented in Figure 1. A total of 2,620 records were retrieved from the electronic databases and five additional articles were found by manually searching reference lists of those records included in the full text screening. Most records were excluded because they did not mention vestibular side effects, did not mention platinum-based chemotherapy, or were *in vitro* or *in vivo* studies. Overall, 75 individual studies were included for data extraction. The sample size of the study participants varied from 1 to 952. The age reported ranged from 11 to 83 years. Study patients had a variety of cancer sites including head and neck cancer, testicular cancer, gynecologic cancer, pulmonary cancer, breast cancer, brain cancer, and other sites. Patients also received concurrent cranial irradiation which may involve areas of inner ear organs in brain, and head and neck cancers (12, 35–38). Platinum-based medication reported in the included studies were cisplatin, carboplatin, oxaliplatin, and non-specified platinum compounds.

### Thematic analysis

From the data extraction and thematic analysis, five themes were defined to determine areas of interest for an overview of platinum-based vestibulotoxicity: (1) objective tests of vestibular and/or balance function, (2) patients' symptoms, (3) physical examination, (4) associated factors such as dosage, pre-existing vestibular loss, and accompanying cochleotoxicity, and (5) general considerations. Results are presented according to these themes.

### Objective tests of vestibular and/or balance function (*N* = 10)

Ten individual studies reported outcomes using one or more objective vestibular or balance function tests (Table 1). Of these, there were multiple publications based on the same study; one reporting a prospective before and after study, plus three corresponding case reports (12, 36–38), and another reporting the same prospective before and after study in two publications (42, 43). Other studies were four prospective before and after studies (13, 39–41), and four cross-sectional studies (35, 44–46). Almost all of these reports focused on toxicities from cisplatin. From these 10 studies, several objective techniques to evaluate vestibular or balance function were reported.

**Table 1:** Summary data of studies that reported objective test results.

**Study no**.	**First author**	**Sample size**	**Population**	**Platinum-based drug and dosage**	**Vestibular function test**	**Balance function test**	**Patients' symptoms**	**Physical examination**	**Concurrent symptoms**
1	Schaefer et al. (12, 36)	24	Adult, head and neck cancer	Cisplatin 100–800 mg/m^2^	4.2% had bilateral decreased caloric response before chemotherapy then absent response after the treatment.	No data	3% had transient dizziness. One patient reported that the symptoms became more problematic in the dark.	No data	3 patients with vestibular symptoms had concurrent sensorineural hearing loss.
2	Black et al. (39)	16	Adult, head and neck cancer	Cisplatin 100–800 mg	31.2% had abnormal rotational test results after completing chemotherapy treatment. 18.8% had new onset or worsening of rotational test results. 12.5% had transient reduction of vestibular function detected by rotational tests.	18.8% had abnormal postural test after completing chemotherapy treatment.	No data	No data	No data
3	Hartwig et al. (40)	74	Adult, testicular and gynecologic cancer	Cisplatin 130–590 mg/m^2^	4.1% had transient abnormal caloric and rotational test	No data	0%	Bedside postural tests, Romberg's test, gait tests, stepping test, and optokinetic test remained normal in all patients	4.1% had hearing loss (Different patients to those with transient vestibular loss).
4	Kobayashi et al. (41)	10	Adult and padiatric, uterus, larynx, orbit, bladder, and bone cancer	Cisplatin 80–550 mg	50% had abnormal caloric test results.20% had abnormal rotational test results.	54.5% had abnormal body sway test.	30% complained of unsteadiness.	70% had spontaneous nystagmus. 60% had positional nystagmus. All patients had normal optokinetic test results.	40% had hearing loss. 50% had tinnitus.
5	Kitsigianis et al. (42, 43)	9	Adult, testicular and pulmonary cancer	Cisplatin 360–800 mg/m^2^	Vestibular autorotation test (VAT) showed decreased VOR gain and increased phase lag.	No data	0%	No data	No data
6	Myers et al. (13)	34	Adult, head and neck cancer	Cisplatin 100–600 mg/m^2^	2.9% had abnormal caloric test results after the treatment. 20.6% had abnormal results rotational test after the treatment. 8.8% had transient reduction in caloric test results. 36.5% had transient VOR gain reduction in rotational test.	No data	0% complained of vertigo or imbalance	No data	58.3% had hearing loss 66.7% had tinnitus
7	Waissbluth et al. (35)	12	Pediatric, brain and liver cancer	Cisplatin 100–800 mg/m^2^Carboplatin 1,000–2,800 mg/m^2^	25% had decreased VOR gain and one of these had overt saccade detected by video head impulse test (vHIT)	No data	41.7% reported recurrent vertigo 16.7% referred disequilibrium	25% had inability to walk in tandem gait 16.6% had gaze-evoked nystagmus	No data
8	Camet et al. (44)	50	Pediatric, brain and other cancer	Cisplatin and carboplatin	No data	6% had abnormal score of Modified Clinical Test of Sensory Interaction on Balance (CTSIB-M)	42% had abnormal score of Pediatric Vestibular Symptom Questionnaire (PVSQ)	28% had abnormal dynamic visual acuity test	No data
9	Miaskowski et al. (45)	623	Adult, breast, colon, lung, ovarian, and other cancer	Platinum compound	No data	Patients with chemotherapy-induced neuropathy had significant worse score on Time Up and Go (TUG) test and the Fullerton Advanced Balance (FAB) test	No data	No data	Patients with neurotoxicites are defined as those who had all toxicities of hearing loss, tinnitus, and neuropathy.
10	Miaskowski et al. (46)	195	Adult, breast, colon, lung, ovarian, and other cancer	Platinum compound	No data	Patients with neurotoxicities side effects had significant worse score on Time Up and Go (TUG) test and the Fullerton Advanced Balance (FAB) test	64% of patients who had chemotherapy-induced neurotoxicities and 14% of patients without those toxicities reported trouble with balance.	No data	Patients with neurotoxicites are defined as those who had all toxicities of hearing loss, tinnitus, and neuropathy.

Objective tests for vestibular function included the caloric test (12, 13, 36, 40, 41), rotational tests (13, 39–41), vestibular autorotation test (VAT) (42, 43), and horizontal video head impulse test (vHIT) (35). Details are given in Table 2. All of these tests detect the horizontal VOR abnormalities. Most studies reported a single vestibular function test such as the caloric test (12, 36), VAT (42, 43), and vHIT (35). Three studies evaluated both caloric and rotational tests (13, 40, 41). The rate of abnormal findings detected by the caloric test after chemotherapy administration varied between 0% (40), 2.9% (13), 4.2% (12, 36), and 50.0% (41). The rate of abnormal findings detected by the rotational test after chemotherapy administration ranged between 0% (40), 20.0% (41), 20.6% (13), and 31.2% (39). Abnormal vestibular function, as detected by the vHIT, was reported in 25.0% of survivors (35). Transient reduction of vestibular function on testing was reported in three studies (13, 39, 40). Bilateral vestibular hypofunction was reported in studies using the caloric test (12, 36) and vHIT (35), whilst some studies did not report which side had the abnormal caloric responses (13, 41). The rotational test and VAT were useful in detecting bilateral vestibular impairment, but the relative contribution could not be discriminated from the results (13, 39, 40, 42, 43).

**Table 2:** Examples of objective tests of vestibular function.

**Test**	**Pathway of testing**	**Procedure**	**Advantages**	**Limitations**
Caloric test	VOR via horizontal SCC at low frequency stimulus (0.002–0.004 Hertz)	Cold and warm water or air are irrigated into the external auditory canal. Nystagmus is detected and the two sides are compared.	- Localization, separate testing of each ear	- Non-physiologic because of relatively low frequency testing - Can induce intense vertigo/dizziness symptoms - Bulky equipment - More problematic in the interpretation of bilateral vestibular dysfunction
Rotational chair test	VOR via horizontal SCC at low-mid frequency stimulus (0.01–0.7 Hertz)	Patient sits in a computerized chair and wears video goggles. Eye movements are recorded during rotation of the chair.	- Useful for bilateral vestibular loss - Can be performed in children	- Lack of localization - Bulky equipment
Active head rotation test (Autorotation test)	VOR via horizontal SCC at mid frequency stimulus (0.5–6 Hertz)	Eye movements are recorded during active head movement in synchrony with audio clicks of different frequencies.	- Rapid assessment - Patients can tolerate the test well without uncomfortable feelings - Portable equipment	- Lack of localization - Compensatory mechanisms during active head movement
Video head impulse test (vHIT)	VOR via all six SCC at high frequency stimulus (4–7 Hertz)	Patient sits on a chair and is instructed to stare at a target on the wall. Equipment to record head movements and video goggles are placed. The quick and unpredictable head turn is carried out by the tester.	- Canal and side specific - Can detect covert saccade (pathologic eye movement during head movement) - Rapid assessment - Most people can tolerate the test well - Portable equipment	- Cannot perform in patients with cervical spine problems - Operator learning curve exists - Expensive
Vestibular Evoked Myogenic Potentials (VEMPs) 1. oVEMP (Ocular) 2. cVEMP (Cervical)	Otolith organs - Utricle - Saccule	Loud sound is presented and muscle activation, either at neck or ocular muscles, is detected through the surface electrodes.	- Rapid assessment - Can be tested in patients with sensorineural hearing loss	- Conductive hearing loss can obliterate VEMPs

*Source (47)*.

Objective tests for balance function included the postural test (39, 41), the Time Up and Go (TUG) test (45, 46), the Fullerton Advanced Balance (FAB) scale (45, 46), and the Modified Clinical Test of Sensory Interaction on Balance (CTSIB-M) (44). Details are given in Table 3. Two of these studies also assessed vestibular dysfunction (39, 41). The abnormal rate of the postural test after completion of chemotherapy treatment was 18.8% (39), and 54.5% (41). Cancer survivors with chemotherapy-induced neuropathy (CIN) had significantly worse scores on both the Time Up and Go (TUG) test and the Fullerton Advanced Balance (FAB) scale (45, 46). Three out of fifty patients (6%) who underwent cancer treatment during their childhood had an abnormal score of CTSIB-M when long-term effects were tested more than 10 years afterwards (44).

**Table 3:** Examples of objective tests of balance function.

**Test**	**Pathway of testing**	**Procedure**	**Advantages**	**Limitations**
Postural test (Posturography)	- Both motor and sensory balance system - Vestibulo-cerebellar reflex (VCR) pathway	Patient is instructed to stand in various conditions such as on a fixed or moving force plate with eyes open or closed.	- Isolate and quantify the principal sensory inputs - Quantitative results	- Limitation in some severely ill people that cannot stand without supports - Expensive - Bulky equipment
Modified Clinical Test of Sensory Interaction on Balance (CTSIB-M)	- Both motor and sensory balance system - Vestibulo-cerebellar reflex (VCR) pathway	Patient is instructed to stand without shoes with feet together and arm crossed for up to 30 seconds in various conditions: solid surface or on foam with eyes open or closed. Time to complete the task is recorded.	- Isolate and quantify the principal sensory inputs - Simple - Screening test	- Less quantitative results
Time Up and Go (TUG) test	Functional body balance by testing performance-based activities	Patient is instructed to stand from an armed chair, walk 10 feet, turn, and return to a seated position. Time to complete the task is recorded.	- Simple - Screening test	- Lack of localization
Fullerton Advanced Balance (FAB) scale	Functional body balance by testing performance-based activities	Patient is instructed to do 10 tasks comprised of standing with feet together and eyes closed, reaching forward to retrieve an object held at shoulder height with an outstretched arm, turning 360 degrees to the right and left, stepping up onto and over a 6-inch bench, tandem walking, standing on one leg, standing on foam with eyes closed, two-footed jumping, walking with head turns, and performing reactive postural control. The ability to complete the tasks is scored.	- Simple - Screening test	- Lack of localization

*Source (47)*.

The testing interval varied amongst studies. Some studies reported testing before each subsequent dose (12, 36, 40, 42, 43), one study tested at variably different time points (39), and some studies did not specify the interval of testing (13, 41). Cross-sectional studies assessed long-term side effects with no availability of baseline comparison (35, 44–46).

### Patients' symptoms (*N* = 74)

Seventy-four studies mentioned some forms of patient-reported symptoms relating to possible vestibular or balance problems. It is difficult to reliably distinguish vestibular from balance dysfunction based on the symptoms alone (8). As a result, symptoms are reported collectively. Terms used by study authors to describe vestibular side effects are often used interchangeably and nonspecifically, for instance, “dizziness” includes “disequilibrium,” “lightheadedness,” and “vertigo” according to the Common Terminology Criteria for Adverse Events (CTCAE) (48). None of the included reports explicitly clarified the definition of these terms which may lead to some degree of ambiguity.

Most studies did not report on the methods used for patient symptom evaluation such as spontaneous reports, patient checklists, interviews or reports by the clinicians. The only study that used a standardized patient-reported outcome questionnaire was the study by Camet (44), which reported 21 out of fifty pediatric cancer survivors (42%) with an abnormal score on the Pediatric Vestibular Symptom Questionnaire (PVSQ).

Regarding the results of those studies with objective vestibular function tests, the rate of reported vestibular symptoms varied from 0% (13, 40, 42, 43) to 41.7% (35). Whilst some vestibular impairment was detected by an objective test, patients did not report any subjective sensation of vestibular disorder (13, 40, 42, 43). Vestibular symptoms reported were transient dizziness (12, 36), unsteadiness (41), and vertigo (35). One patient had compensated well for his vestibular loss, except in the dark (36). Patients' symptoms were not reported in one study despite a high rate of abnormal vestibular test results (39).

Many examples were found of studies where patients complained of symptoms that were potentially due to vestibular side effects, but without verification by objective measurement. Studies in cancer treatment involving multiple medications including platinum-based drugs have reported non-specific balance symptoms such as dizziness, vertigo, ataxia, balance problems, and gait disturbance. A recent multi-center study of 952 testicular cancer survivors receiving cisplatin-based chemotherapy stated 9.3% overall new cases of dizziness, vertigo or balance problems (49).

### Physical examination (*N* = 4)

Physical examinations were reported in four studies (35, 40, 41, 44). In the study by Kobayashi et al. (41), there were reports of spontaneous nystagmus (nystagmus in resting position without any stimulus) in seven out of 10 patients (70%) and positional nystagmus (nystagmus in a specific head position) in 6 out of 10 patients (60%). All three patients that complained of dizziness had spontaneous nystagmus and two of them had positional nystagmus. However, not all patients who had spontaneous nystagmus complained of dizziness. The optokinetic tests, Romberg's tests, gait tests and stepping tests remained normal in all 74 patients (40). The rate of abnormal results of dynamic visual acuity (DVA) as a vestibular screening test was 28% (44). Three out of twelve patients (25%) diagnosed with brain cancer had an inability to walk in tandem gait and two of them (16.6%) had gaze-evoked nystagmus (35).

### Associated factors (*N* = 8)

Some associated factors of vestibulotoxicity mentioned in the literature were dosage, pre-existing vestibular dysfunction, and accompanying cochleotoxicity. Vestibular function was reported to have declined with increasing dose of cisplatin within each patient (39, 42, 43). However, the data reviewed did not allow the identification of specific cumulative dose thresholds associated with vestibular or balance impact. There is some evidence to suggest that subjects with pre-existing loss of vestibular function are more likely to get cisplatin vestibular toxicity. Two of five patients (40%) with prior abnormal vestibular function had additional vestibular loss after the cessation of the treatment, whilst one out of nine patients (11.1%) with prior normal vestibular function developed vestibular toxicity after chemotherapy (39). The only report of the histological verification showed severe loss of hair cells in peripheral vestibular sensory organs, including a crystalline spherical concretion composed of calcium carbonate in the left posterior semicircular duct, which is compatible with a positive finding of benign paroxysmal positional vertigo (BPPV) (37, 38). This could be either the underlying cause of dizziness or an incidental finding.

Vestibular toxicity defined by an abnormal vestibular function test was accompanied by cochlear toxicity (either hearing impairment or tinnitus) in three studies (12, 35, 36, 41), whilst no hearing loss was found in three patients with reduced caloric and rotatory response in one report (40), and no hearing status was stated in the other studies (13, 39, 42, 43). None of these studies stated the temporal correlation of vestibular and cochlear symptoms.

### General considerations

By pooling together details of the data items, some of the general considerations about development of relevant literature, and treatment modalities for cancer emerge from the literature.

#### Development of relevant literature

Some preliminary studies were conducted in the 1980s followed by few studies after that. Whilst the number of publications has increased since the 2000s, the majority of these mentioned symptoms that were possibly due to vestibular side effects. A few studies explored vestibular-related long-term effects of chemotherapy recently indicating a growing awareness and interest from the research community of vestibular toxicity of platinum-based chemotherapy.

#### Treatment modalities for cancer

Treatment options are determined by cancer sites and staging. Multimodality treatment and combination of cytotoxic medications are common for cancer treatment. Patients also received concurrent cranial irradiation, which may involve areas of inner ear organs especially in brain, and head and neck cancers (12, 35–38). Vincristine, etoposide and bleomycin have been co-administered for cancer treatment (42, 43). One of three symptomatic patients also received an aminoglycoside antibiotic, amikacin (35), which is a known vestibulotoxic medication (17). Moreover, cisplatin, carboplatin, and oxaliplatin may have different degrees of vestibulotoxicity due to drug components, pharmacodynamics, and pharmacokinetics (50).

## Discussion

This scoping review explored issues related to the vestibular effects of platinum-based chemotherapy in order to identify established knowledge and research gaps which warrant further research.

### Conclusions based on established knowledge

Evaluation of vestibulotoxicity in the existing literature included objective tests, findings of physical examination, and patient reported symptoms. Tests for evaluation of vestibulotoxicity in previous publications have been largely focused on low to mid frequencies of horizontal semicircular canal testing, utilizing the caloric test, rotational tests, and the VAT. VHIT, a relatively new technique, was used to test function of the horizontal semicircular canal at higher frequency stimulation in one recent study (35). The existing information of vestibular toxicity in the literature examined only the horizontal semicircular canal which is one of five peripheral vestibular sensory organs. This may be due to the feasibility of testing all potentially affected organs, or the fact that sensory cells in the semicircular canals are more sensitive to toxic medication than those in the otolith organs (51).

Vestibular function loss may not be recognized until the patient loses other cues from vision and somatosensory such as when walking in the dark or develops concomitant peripheral neuropathy. Balance function tests such as posturography, testing overall balance system function in various conditions, can provide additional information on balance in real life and dynamic situations. Subclinical vestibular toxicity is a concern because its effect may only be evident in patients with other impaired sensory inputs, previous vestibular problems, or it may lead to earlier onset of age-related vestibular impairment (52).

The distinction between “objective tests of clinical signs” that can be measured by the clinicians and “subjective clinical symptoms” that can only be reported by the patients should be emphasized. The existing literature showed that objective test findings and patient symptoms did not always correspond to one another. Multiple studies reported abnormal vestibular function tests in asymptomatic patients (13, 40, 42, 43), therefore it is quite clear that clinicians cannot rely solely on symptoms to detect vestibular toxicity. It is not surprising that most patients did not have intense symptoms due to the potential bilateral symmetrical insidious nature of ototoxic medication. Symptoms such as vertigo, dizziness, headache, nausea, double vision, photophobia, ataxia, and light-headedness have previously been reported in patients with bilateral vestibular impairment (53, 54). Balance symptoms such as dizziness and unsteadiness are associated with vestibular dysfunction; however, the relationship between vestibulotoxicity and non-specific balance symptoms may not always be clear.

Dizziness and other balance problems can be difficult to describe for the patient and to categorize by the physician. Hence, clarification of the terminology used is important for the interpretation. These non-specific symptoms of balance problems might automatically be associated with cancer diseases and general deconditioning of patients. Comorbid factors such as peripheral neuropathy, fatigue, and anemia, including brain pathology in brain cancer patients can cause some elements of postural unsteadiness in cancer patients. Clinical symptoms are often underappreciated by both patients and clinicians. For example, vestibular toxicity may be a subtle complaint presenting as nausea, vomiting, or dizziness, which are not uncommon in cancer patients receiving chemotherapy.

Known risk factors of cochlear ototoxicity are aging, the cumulative dose of chemotherapy, poor renal function, and co-administration of other ototoxic medications (55). Possible potential risk factors of vestibular toxicity identified in the literature are cumulative dose and pre-existing vestibular loss. Decreased vestibular function occurred as cumulative dose of cisplatin increased, which supports the assumption of dose-related response (39, 42, 43). There is some evidence to tentatively support that patients with pre-existing loss of vestibular function are prone to cisplatin vestibular toxicity, for instance, the additional loss of vestibular impairment also occurred in patients with previous abnormal vestibular function (12, 36, 39).

### Knowledge gaps

The rate of abnormal vestibular function test findings associated with platinum-based chemotherapy in the existing literature varied from 0 to 50% after chemotherapy treatment and 4.3–36.5% during chemotherapy pathway. These should be viewed as preliminary reports due to variable methodology, limited reported information and the relatively small number of patients. Therefore, the incidence and prevalence of vestibular abnormality after platinum-based chemotherapy warrant further research.

None of the studies evaluated vestibular function using vHIT to test all six semicircular canals, nor vestibular evoked myogenic potentials (VEMPs) to test otolith organs. A single test may not be a good indicator of the true vestibular function and findings from a comprehensive vestibular test battery have not been yet reported. Limitations of vestibular and balance function tests are summarized in Tables 2, 3. The current review gleaned sparse information and details on the procedure of the physical examination. Baseline testing is required to evaluate prior vestibular impairment and to detect subtle changes. Based on the existent information, data on affected sites of lesions in vestibular end organs, and the impact on higher frequency function associated with vestibulotoxicity in clinical studies is still lacking.

Most of the included reports did not mention the methods used for patient symptom evaluation. The approaches utilized in detecting or monitoring adverse effects, such as spontaneous reporting, patient checklist, questionnaire, diary, systematic survey of patients or report by the investigators, are known to have an influence on the frequencies of adverse effects identified (56). For example, passive monitoring based on spontaneous reports might yield lower rates of adverse events, while active surveillance using specific questioning could find a higher rate.

The association between cochlear impairment and vestibular toxicity is still unknown. Cochlear function deficit is more frequently described in the literature (4), potentially because it is more common, or it is recognized earlier and before vestibular impairment, symptoms of which tend to occur after complete bilateral loss or asymmetrical loss (14).

BPPV is common in the general population, including subjects receiving ototoxic drugs (57). BPPV presenting in subjects receiving ototoxic drugs may complicate the clinical identification of ototoxicity and obscure the clinical decision. Nevertheless, the possible role of ototoxicity in the pathophysiology of BPPV was not clear from the literature.

A combination of treatments is common in cancer treatment so evaluating the relationship between platinum-based chemotherapy and vestibulotoxicity may not be straightforward. Vestibulotoxic medications such as aminoglycoside antibiotics (17) are sometimes used to cure infections in cancer patients. Although most chemotherapeutic agents are not classified as ototoxic medication, vincristine (58) and bleomycin (59) have been shown to cause cochlear hair cell damage in *in vivo* studies. It has also been postulated that platinum-based chemotherapy and radiotherapy have a combination of effects on cochleotoxicity (60, 61). Currently, there is limited information not only on platinum-based chemotherapy but also other treatment modalities and their combinations which should be taken into account in data interpretation.

### Limitations of the study

At present, there are methodological limitations in the published literature about vestibular effects of ototoxic medications. Some limitations of the current review are that a significant portion of included studies reported limited information, and only publications in the English language were included; thus, language bias may have occurred.

## Conclusion

A number of studies reported significant evidence of cisplatin vestibular toxicities with objective tests, although not always corroborated by patient symptoms. Multiple studies also reported non-specific imbalance symptoms which are possible complaints of vestibular dysfunction; however, the nature of these symptoms is unclear without any objective test. There was very limited clinical research data to date on the vestibular side effects of platinum-based chemotherapy in terms of incidence, evaluation and impact on health-related quality of life of the patients. The data of physical examination was reported in only a few studies and was not comprehensive. The current evidence was based solely on the horizontal semicircular canal evaluation using the caloric tests or rotational tests; therefore, the information of vertical semicircular canals and otolith organs is still lacking.

In conclusion, this scoping review summarizes the current research findings that vestibular toxicity needs more attention and emphasizes the need for future high-quality studies in this field. Comprehensive evaluation of vestibular toxicities, identifying risk factors such as cumulative dose, pre-existing abnormal vestibular function and aging, including the correlation with cochlear toxicity and peripheral neuropathy warrant further research. Another challenge tends toward which test would be the most sensitive and appropriate for early detection and monitoring changes before, during and after platinum-based chemotherapy treatment.

## Author contributions

PP conducted the review and wrote the manuscript. DB conducted the review and helped revise the manuscript. JT, SP, RG, PMP, DH, and AK conducted the study selection and helped revise the manuscript.

## Data availability statement

All datasets for this study are included in the manuscript and the Supplementary Files.

### Conflict of interest statement

The authors declare that the research was conducted in the absence of any commercial or financial relationships that could be construed as a potential conflict of interest.
