# Supplementary material for: Vestibulotoxicity Associated With Platinum-Based Chemotherapy in Survivors of Cancer: A Scoping Review
Source: Front Oncol. 2018 Sep 25;8:363. doi: 10.3389/fonc.2018.00363 (PMC6167545; doi:10.3389/fonc.2018.00363)
Supplement: Supplementary file 1 [file Table_1.DOCX]

Supplementary Material

Vestibulotoxicity associated with platinum-based chemotherapy in survivors of cancer: a scoping review

Pattarawadee Prayuenyong, John A Taylor, Stephanie E Pearson, Rachel Gomez, Poulam M Patel, Deborah A Hall, Anand V Kasbekar, and David M Baguley

*** Correspondence:** Dr Pattarawadee Prayuenyong: msxpp4@nottingham.ac.uk

# Appendix 1: Search strategies

**A. CENTRAL**

([mh Carboplatin] OR [mh Cisplatin] OR [mh "Organoplatinum Compounds"] OR [mh "Platinum Compound"] OR [mh Platinum] OR (Biocisplatinum OR Blastocarb OR Carboplat* OR Carbosin OR Carbotec OR CBDCA OR CDDP OR Cisplatin* OR Cisplatinum OR Cisplatyl OR Diamminedichloroplatinum OR Dichlorodiammineplatinum OR Ercar OR JM OR "JM8" OR "JM 8" OR Nealorin OR Nedaplatin OR Neocarbo OR "NOV002" OR "NSC 119875" OR "NSC119875" OR "NSC241240" OR "NSC 241240" OR Organoplatinum* OR Oxaliplatin* OR Paraplatin OR Platidiam OR Platin* OR Ribocarbo):ti,ab) AND ([mh "Accidental Falls"] OR [mh Ataxia] OR [mh Dizziness] OR [mh "Caloric Tests"] OR [mh "Ear, Inner"] OR [mh Electronystagmography] OR [mh "Head Impulse Test"] OR [mh "Labyrinth Diseases"] OR [mh "Nystagmus, Pathologic"] OR [mh "Postural Balance"] OR [mh "Reflex, Vestibulo-Ocular"] OR [mh Vertigo] OR [mh "Vestibular Diseases"] OR [mh "Vestibular Function Tests"] OR [mh "Vestibule, Labyrinth"] OR (Atax* OR Barany* Test* OR Caloric Test* OR Coordination Impairment* OR "Coordination Lack" OR Disequilibrium OR Dizz* OR Dyscoordination OR Dysequilibrium OR Dyssynergia OR Electronystagmogra* OR Fall OR Falling OR Falls OR Gidd* OR Head Heave Test OR Head Impulse Test OR Head Thrust Test OR Imbalance OR Incoordination* OR Inner Ear* OR Internal Ear* OR Labyrinth* OR "Lack of Coordination" OR Light Headedness OR Lightheadedness OR Musculoskeletal Equilibrium OR Nystagm* OR Orthostasis OR Oscillopsia OR Postural Balance OR Postural Equilibrium OR Rubral Tremor* OR Spinning Sensation* OR Vertigo OR Vestib*):ti,ab)

**B. ClinicalTrials.Gov**

Study Type: Interventional Studies

Study Results: Studies With Results

Status: Studies: Suspended; Terminated; Completed; Withdrawn

Intervention / Treatment: Carboplatin OR Cisplatin OR Organoplatinum OR Oxaliplatin

Outcome Measures: Adverse OR Side Effect OR Side Effects OR Ataxia OR Dizziness OR Labyrinth OR Nystagmus OR Vertigo OR Vestibular OR Fall OR Falls OR Imbalance OR Inner Ear OR Equilibrium

**C. EMBASE**

1. Carboplatin/ OR Cisplatin/ OR Cisplatin Derivative/ OR Oxaliplatin/ OR Platinum Complex/ OR Platinum Derivative/ OR Platinum/ OR (Biocisplatinum OR Blastocarb OR Carboplat* OR Carbosin OR Carbotec OR CBDCA OR CDDP OR Cisplatin* OR Cisplatinum OR Cisplatyl OR Diamminedichloroplatinum OR Dichlorodiammineplatinum OR Ercar OR JM OR "JM8" OR "JM 8" OR Nealorin OR Nedaplatin OR Neocarbo OR "NOV002" OR "NSC 119875" OR "NSC119875" OR "NSC241240" OR "NSC 241240" OR Organoplatinum* OR Oxaliplatin* OR Paraplatin OR Platidiam OR Platin* OR Ribocarbo).ti,ab.
2. Adverse Drug Reaction/ OR (Adverse OR Side Effect* OR Toxic*).ti,ab.
3. Falling/ OR Ataxia/ OR Dizziness/ OR Caloric Vestibular Test/ OR Inner Ear/ OR Electronystagmography/ OR Head Impulse Test/ OR Exp Inner Ear Disease/ OR Nystagmus/ OR Body Equilibrium/ OR Vestibuloocular Reflex/ OR Vertigo/ OR Exp Vestibular Disorder/ OR Exp Vestibular Function/ OR Exp Vestibular Test/ OR Exp Vestibular Labyrinth/ OR Vestibular Nystagmus/ OR (Atax* OR Barany* Test* OR Body Sway OR Caloric Test* OR Coordination Impairment* OR "Coordination Lack" OR Disequilibrium OR Dizz* OR Dyscoordination OR Dysequilibrium OR Dyssynergia OR Electronystagmogra* OR Fall OR Falling OR Falls OR Gidd* OR Head Heave Test OR Head Impulse Test OR Head Thrust Test OR Imbalance OR Incoordination* OR Inner Ear* OR Internal Ear* OR Labyrinth* OR "Lack of Coordination" OR Light Headedness OR Lightheadedness OR Equilibrium OR Nystagm* OR Oculovestib* OR Orthostasis OR Oscillopsia OR Postural Balance OR Rubral Tremor* OR Spinning Sensation* OR Vertigo OR Vestib*).ti,ab.
4. AND/1-3
5. Carboplatin/ae, to [Adverse Drug Reaction, Drug Toxicity]
6. Cisplatin/ae, to [Adverse Drug Reaction, Drug Toxicity]
7. Cisplatin Derivative/ae, to [Adverse Drug Reaction, Drug Toxicity]
8. Oxaliplatin/ae, to [Adverse Drug Reaction, Drug Toxicity]
9. Platinum Complex/ae, to [Adverse Drug Reaction, Drug Toxicity]
10. Platinum Derivative/ae, to [Adverse Drug Reaction, Drug Toxicity]
11. Platinum/ae, to [Adverse Drug Reaction, Drug Toxicity]
12. OR/5-11
13. Ataxia/si [Side Effect]
14. Dizziness/si [Side Effect]
15. Inner Ear Disease/si [Side Effect]
16. Nystagmus/si [Side Effect]
17. Vertigo/si [Side Effect]
18. Vestibular Disorder/si [Side Effect]
19. Vestibular Nystagmus/si [Side Effect]
20. OR/13-19
21. AND/3, 12
22. AND/1, 20
23. 4 OR 21 OR 22
24. Exp Animals/ OR Exp Invertebrate/ OR Animal Experiment/ OR Animal Model/ OR Animal Tissue/ OR Animal Cell/ OR Nonhuman/
25. Human/ OR Normal Human/ OR Human Cell/
26. AND/24-25
27. 24 NOT 26
28. 23 NOT 27
29. Limit 28 to Conference Abstracts
30. 28 NOT 29
31. Limit 30 to (English Language and EMBASE and Exclude MEDLINE Journals and YR="1978 -Current")

**D. International Clinical Trials Registry Platform**

Cancer in the Condition

Platinum AND (Carboplatin OR Cisplatin OR Oxaliplatin) in the Intervention

Recruitment Status is ALL

**E. International Pharmaceutical Abstracts**

1. Carboplatin/ OR Cisplatin/ OR Organoplatinum Compounds/ OR Platinum Compound/ OR Platinum/ OR (Biocisplatinum OR Blastocarb OR Carboplat* OR Carbosin OR Carbotec OR CBDCA OR CDDP OR Cisplatin* OR Cisplatinum OR Cisplatyl OR Diamminedichloroplatinum OR Dichlorodiammineplatinum OR Ercar OR JM OR "JM8" OR "JM 8" OR Nealorin OR Nedaplatin OR Neocarbo OR "NOV002" OR "NSC 119875" OR "NSC119875" OR "NSC241240" OR "NSC 241240" OR Organoplatinum* OR Oxaliplatin* OR Paraplatin OR Platidiam OR Platin* OR Ribocarbo).ti,ab.
2. Accidental Falls/ OR Ataxia/ OR Dizziness/ OR Caloric Tests/ OR Ear, Inner/ OR Electronystagmography/ OR Head Impulse Test/ OR Labyrinth Diseases/ OR Nystagmus, Pathologic/ OR Postural Balance/ OR Reflex, Vestibulo-Ocular/ OR Vertigo/ OR Vestibular Diseases/ OR Vestibular Function Tests/ OR Vestibule, Labyrinth/ OR (Atax* OR Barany* Test* OR Caloric Test* OR Coordination Impairment* OR "Coordination Lack" OR Disequilibrium OR Dizz* OR Dyscoordination OR Dysequilibrium OR Dyssynergia OR Electronystagmogra* OR Fall OR Falling OR Falls OR Gidd* OR Head Heave Test OR Head Impulse Test OR Head Thrust Test OR Imbalance OR Incoordination* OR Inner Ear* OR Internal Ear* OR Labyrinth* OR "Lack of Coordination" OR Light Headedness OR Lightheadedness OR Musculoskeletal Equilibrium OR Nystagm* OR Orthostasis OR Oscillopsia OR Postural Balance OR Postural Equilibrium OR Rubral Tremor* OR Spinning Sensation* OR Vertigo OR Vestib*).ti,ab.
3. 1 AND 2
4. Limit 3 to (English Language and Human and YR="1978 -Current")

**F. MEDLINE**

1. Carboplatin/ OR Cisplatin/ OR Organoplatinum Compounds/ OR Platinum Compound/ OR Platinum/ OR (Biocisplatinum OR Blastocarb OR Carboplat* OR Carbosin OR Carbotec OR CBDCA OR CDDP OR Cisplatin* OR Cisplatinum OR Cisplatyl OR Diamminedichloroplatinum OR Dichlorodiammineplatinum OR Ercar OR JM OR "JM8" OR "JM 8" OR Nealorin OR Nedaplatin OR Neocarbo OR "NOV002" OR "NSC 119875" OR "NSC119875" OR "NSC241240" OR "NSC 241240" OR Organoplatinum* OR Oxaliplatin* OR Paraplatin OR Platidiam OR Platin* OR Ribocarbo).ti,ab.
2. Long Term Adverse Effects/ OR "Drug-Related Side Effects and Adverse Reactions"/ OR (Adverse OR Side Effect* OR Toxic*).ti,ab.
3. Accidental Falls/ OR Ataxia/ OR Dizziness/ OR Caloric Tests/ OR Ear, Inner/ OR Electronystagmography/ OR Head Impulse Test/ OR Exp Labyrinth Diseases/ OR Nystagmus, Pathologic/ OR Postural Balance/ OR Reflex, Vestibulo-Ocular/ OR Vertigo/ OR Exp Vestibular Diseases/ OR Exp Vestibular Function Tests/ OR Exp Vestibule, Labyrinth/ OR (Atax* OR Barany* Test* OR Caloric Test* OR Coordination Impairment* OR "Coordination Lack" OR Disequilibrium OR Dizz* OR Dyscoordination OR Dysequilibrium OR Dyssynergia OR Electronystagmogra* OR Fall OR Falling OR Falls OR Gidd* OR Head Heave Test OR Head Impulse Test OR Head Thrust Test OR Imbalance OR Incoordination* OR Inner Ear* OR Internal Ear* OR Labyrinth* OR "Lack of Coordination" OR Light Headedness OR Lightheadedness OR Musculoskeletal Equilibrium OR Nystagm* OR Orthostasis OR Oscillopsia OR Postural Balance OR Postural Equilibrium OR Rubral Tremor* OR Spinning Sensation* OR Vertigo OR Vestib*).ti,ab.
4. AND/1-3
5. Carboplatin/ae, ct, to [Adverse Effects, Contraindications, Toxicity]
6. Cisplatin/ae, ct, to [Adverse Effects, Contraindications, Toxicity]
7. Organoplatinum Compounds/ae, ct, to [Adverse Effects, Contraindications, Toxicity]
8. Platinum Compounds/ae, to [Adverse Effects, Toxicity]
9. Platinum/ae, to [Adverse Effects, Toxicity]
10. OR/5-9
11. AND/3, 10
12. Ataxia/ci [Chemically Induced]
13. Dizziness/ci [Chemically Induced]
14. Ear, Inner/de [Drug Effects]
15. Labyrinth Diseases/ci [Chemically Induced]
16. Nystagmus, Pathologic/ci, de [Chemically Induced, Drug Effects]
17. Postural Balance/de [Drug Effects]
18. Reflex, Vestibulo-Ocular/de [Drug Effects]
19. Vertigo/ci [Chemically Induced]
20. Vestibular Diseases/ci [Chemically Induced]
21. Vestibule, Labyrinth/de [Drug Effects]
22. OR/12-21
23. AND/1, 22
24. OR/4, 11, 23
25. (Animals NOT (Humans and Animals)).sh.
26. 24 NOT 25
27. Limit 26 to (English language and yr="1978 -Current")

**G. ProQuest Dissertations & Theses A&I‎**

(ti(Biocisplatinum OR Blastocarb OR Carboplat* OR Carbosin OR Carbotec OR CBDCA OR CDDP OR Cisplatin* OR Cisplatinum OR Cisplatyl OR Diamminedichloroplatinum OR Dichlorodiammineplatinum OR Ercar OR JM OR "JM8" OR "JM 8" OR Nealorin OR Nedaplatin OR Neocarbo OR "NOV002" OR "NSC 119875" OR "NSC119875" OR "NSC241240" OR "NSC 241240" OR Organoplatinum* OR Oxaliplatin* OR Paraplatin OR Platidiam OR Platin* OR Ribocarbo) OR ab(Biocisplatinum OR Blastocarb OR Carboplat* OR Carbosin OR Carbotec OR CBDCA OR CDDP OR Cisplatin* OR Cisplatinum OR Cisplatyl OR Diamminedichloroplatinum OR Dichlorodiammineplatinum OR Ercar OR JM OR "JM8" OR "JM 8" OR Nealorin OR Nedaplatin OR Neocarbo OR "NOV002" OR "NSC 119875" OR "NSC119875" OR "NSC241240" OR "NSC 241240" OR Organoplatinum* OR Oxaliplatin* OR Paraplatin OR Platidiam OR Platin* OR Ribocarbo)) AND (ti(Atax* OR Barany* Test* OR Caloric Test* OR Coordination Impairment* OR "Coordination Lack" OR Disequilibrium OR Dizz* OR Dyscoordination OR Dysequilibrium OR Dyssynergia OR Electronystagmogra* OR Fall OR Falling OR Falls OR Gidd* OR Head Heave Test OR Head Impulse Test OR Head Thrust Test OR Imbalance OR Incoordination* OR Inner Ear* OR Internal Ear* OR Labyrinth* OR "Lack of Coordination" OR Light Headedness OR Lightheadedness OR Musculoskeletal Equilibrium OR Nystagm* OR Orthostasis OR Oscillopsia OR Postural Balance OR Postural Equilibrium OR Rubral Tremor* OR Spinning Sensation* OR Vertigo OR Vestib*) OR ab(Atax* OR Barany* Test* OR Caloric Test* OR Coordination Impairment* OR "Coordination Lack" OR Disequilibrium OR Dizz* OR Dyscoordination OR Dysequilibrium OR Dyssynergia OR Electronystagmogra* OR Fall OR Falling OR Falls OR Gidd* OR Head Heave Test OR Head Impulse Test OR Head Thrust Test OR Imbalance OR Incoordination* OR Inner Ear* OR Internal Ear* OR Labyrinth* OR "Lack of Coordination" OR Light Headedness OR Lightheadedness OR Musculoskeletal Equilibrium OR Nystagm* OR Orthostasis OR Oscillopsia OR Postural Balance OR Postural Equilibrium OR Rubral Tremor* OR Spinning Sensation* OR Vertigo OR Vestib*))Limits applied

Narrowed by: Language: English

Limited to 1978 to 2018

**H. Science Citation Index-Expanded**

TITLE: (Biocisplatinum OR Blastocarb OR Carboplat* OR Carbosin OR Carbotec OR CBDCA OR CDDP OR Cisplatin* OR Cisplatinum OR Cisplatyl OR Diamminedichloroplatinum OR Dichlorodiammineplatinum OR Ercar OR JM OR "JM8" OR "JM 8" OR Nealorin OR Nedaplatin OR Neocarbo OR "NOV002" OR "NSC 119875" OR "NSC119875" OR "NSC241240" OR "NSC 241240" OR Organoplatinum* OR Oxaliplatin* OR Paraplatin OR Platidiam OR Platin* OR Ribocarbo) AND TOPIC: (Atax* OR Barany* Test* OR Caloric Test* OR Coordination Impairment* OR "Coordination Lack" OR Disequilibrium OR Dizz* OR Dyscoordination OR Dysequilibrium OR Dyssynergia OR Electronystagmogra* OR Fall OR Falling OR Falls OR Gidd* OR Head Heave Test OR Head Impulse Test OR Head Thrust Test OR Imbalance OR Incoordination* OR Inner Ear* OR Internal Ear* OR Labyrinth* OR "Lack of Coordination" OR Light Headedness OR Lightheadedness OR Musculoskeletal Equilibrium OR Nystagm* OR Orthostasis OR Oscillopsia OR Postural Balance OR Postural Equilibrium OR Rubral Tremor* OR Spinning Sensation* OR Vertigo OR Vestib*)

Timespan: 1978-2017. Indexes: SCI-EXPANDED

**I. TOXLINE**

(Biocisplatinum OR Blastocarb OR Carboplatin OR Carbosin OR Carbotec OR CBDCA OR CDDP OR Cisplatin OR Cisplatinum OR Cisplatyl OR Diamminedichloroplatinum OR Dichlorodiammineplatinum OR Ercar OR Nealorin OR Nedaplatin OR Neocarbo OR Organoplatinum OR Oxaliplatin OR Paraplatin OR Platidiam OR Platinum OR Ribocarbo) AND (Ataxia OR Barany OR Caloric Test OR Coordination OR Disequilibrium OR Dizziness OR Dyscoordination OR Dysequilibrium OR Dyssynergia OR Fall OR Falls OR Imbalance OR Incoordination OR Ear OR Labyrinth OR Headedness OR Lightheadedness OR Equilibrium OR Nystagmus OR Orthostasis OR Oscillopsia OR Balance OR Equilibrium OR Vertigo OR Vestibular) AND 1978:2018 [yr] AND ( eng [la] ) AND NOT PubMed [org] AND NOT pubdart [org]

**J. Grey Literature**

Database of Adverse Event Notifications via Medicines Safety Update [Australia]

Oxaliplatin AND Ear and labyrinth disorders 1978-01-01 to 2017-08-16 [4 Cases]

Carboplatin AND Ear and labyrinth disorders 1978-01-01 to 2017-08-16 [12 Cases]

Cisplatin AND Ear and labyrinth disorders 1978-01-01 to 2017-08-16 [49 Cases]

Drug Safety Update [UK]

Search Carboplatin AND Therapeutic Area: Cancer: 0

Search Cisplatin AND Therapeutic Area: Cancer: 1

Search Oxaliplatin AND Therapeutic Area: Cancer: 5

European Public Assessment Reports via European Medicines Agency [Europe]

Carboplatin: No Results

Cisplatin: No Results

Oxaliplatin: No Results

Medwatch via Food and Drug Administration (FDA) [USA]

Carboplatin: 1 Report

Cisplatin: No Report

Oxaliplatin No Report

Vigibase via http://www.vigiaccess.org/

Carboplatin Adverse Drug Reactions (ADRs) Ear and Labyrinth Disorders (478 Cases)

Cisplatin Adverse Drug Reactions (ADRs) Ear and Labyrinth Disorders (1281 Cases)

Oxaliplatin Adverse Drug Reactions (ADRs) Ear and Labyrinth Disorders (255 Cases)
